# Stress-informed parametric shape adaptive patient-specific implant design in computer-assisted jaw reconstruction

**DOI:** 10.3389/fonc.2026.1966850

**Published:** 2026-10-16

**Authors:** Ankit Nayak, Yu-Xiong Su, Jane Jingya Pu

**Affiliations:** 1 School of Advanced Engineering, University of Petroleum and Energy Studies (UPES), Dehradun, India; 2 Division of Oral and Maxillofacial Surgery, Faculty of Dentistry, The University of Hong Kong, Hong Kong, Hong Kong SAR, China

**Keywords:** additive manufacturing, computer-aided implant design, finite element analysis, mandibular reconstruction, patient-specific implant, stress-adaptive design, titanium alloy

## Abstract

**Introduction:**

Reconstructing the jaw after tumor removal or trauma increasingly relies on custom 3D-printed titanium plates. This customization applies to the implant's shape. It usually does not apply to the internal structure. The width and thickness of the plate are still determined by the designer, based on experience and clinical observation. The critical design parameters of the PSI govern its mechanical performance. Hence, if the design could analyze these parameters and adjust them accordingly, it could help avoid plate fracture. This study introduces a parametric-design approach that closes this gap.

**Methods:**

The proposed algorithm addresses these challenges by using stress data derived from finite element analysis (FEA) to selectively reinforce only the high-stress regions of a reconstruction plate, rather than uniformly reinforcing the entire plate. The reinforcement is smoothly integrated into the plate’s geometry to prevent abrupt changes in cross-section that could create stress concentrations. The approach was applied to a baseline plate at two regions of elevated stress.

**Results and discussion:**

Plate A reinforced only the primary zone; this reduced peak stress by 36.0% and strain by 43.8% in that zone, the largest reduction observed across both design variants, and also lowered stress and strain in the second, unmodified zone by 12.7% and 14.3%, respectively. Plate B reinforced both zones; this further reduced stress and strain at the second zone by approximately 30.6% and 33.3%, respectively, and reduced strain at the primary zone by approximately 40.6% relative to baseline. Relative to Plate A, Plate B showed a slightly higher stress at the first zone; this stress remained well below the original baseline value. This work shows that patient-specific implant geometry can be shaped directly by biomechanical feedback. This algorithm offers an efficient computational workflow, enabling implants that are not only anatomically tailored but also mechanically optimized as per given loading conditions.

## 1 Introduction

Computer-assisted surgery (CAS) is the current state of the art for jaw reconstruction. It combines three-dimensional virtual surgical planning (VSP) with computer-aided design and manufacturing (CAD/CAM).This integration enables the precise execution of the surgical plan. CAS navigation systems support execution. It also uses patient-specific 3D-printed implants (PSIs). (1). In cases of segmental mandibulectomy for oncological or other destructive diseases, a vascularized free fibula flap (FFF) remains the well-established reconstructive approach (2, 3). In this reconstruction surgery, the virtual plan is transferred to the operating theater (OT) using patient-specific cutting guides and patient-specific implants (4). By reducing freehand contouring and repeated intraoperative measurement, CAS increases the predictability of reconstruction (5). CAS also allows the PSI to maintain the planned three-dimensional position of the fibular segments relative to the residual mandible during fixation (6). Clinical data further indicate that custom fixation systems achieve complication rates comparable to standard reconstruction plates, supporting their use in complex mandibular reconstruction (7).

However, despite this high geometric accuracy in restoring anatomical form (5, 8), the design of the PSI itself is still primarily based on the surgeon’s or designer’s experience and conventional CAD workflows. It largely lacks personalized FEA data to inform the plate’s structural dimensions. Most automated design pipelines aim to create anatomically conforming geometries (9). However, they often do not customize the local cross-sectional thickness or width to match the specific mechanical environment that the implant will encounter. This environment includes cyclic loads transmitted through the mandible in the healed area and/or the patient-specific implant (PSI) during chewing and condylar movement (10, 11).

This experience-driven, geometry-only approach to plate design may carry potential biomechanical risk. This is because uniform-thickness and uniform-width plates reflect two competing design tendencies. Overly thick plates have been associated with stress shielding (12). This has been reported to be potentially associated with impaired bone healing. It may also be associated with increased risk of implant exposure through overlying soft tissue. It has also been associated with radiation scattering (13) during post-operative radiotherapy. Conversely, plates that are too thin have been reported to be associated with a higher risk of fracture. This, in turn, has been associated with bone non-union or mal-union in some reports (14). Stress in the PSI accumulates disproportionately near sharp edges, notches, and screw holes (11). Because of this, a single uniform cross-section may potentially be structurally inefficient.

Therefore, there is an urgent clinical and engineering need for plates that are individually designed using patient-specific FEA, in which local geometry is adaptively scaled according to the predicted stress distribution rather than being fixed *a priori*. While FEA is now commonly used to evaluate the biomechanical performance of mandibular reconstruction plates after the fact (15), its direct integration into an automated, closed-loop geometry-generation process remains largely unexplored (16).

Most existing PSI workflows primarily emphasize anatomical customization. This includes conformity to the patient-specific anatomy and appropriate positioning of fixation features. Structural parameters such as local plate width and thickness, however, are often prescribed from parameter studies or designer-selected values. Some studies have used FEA to investigate the effects of parameters such as plate thickness and screw configuration (17). Comparatively less attention, however, has been given to directly coupling local FEA-derived stress concentrations with automated, spatially localized modification of the plate cross-section. Topology optimization addresses structural customization more directly. It does so by iteratively redistributing material within a prescribed design domain using sensitivity-based optimization methods (18). The resulting geometry, however, may subsequently require redesign or verification to satisfy manufacturing and clinical constraints (19). Parametric design approaches, in contrast, provide an efficient means of generating patient-specific geometry (20). Conventional implementations, however, commonly prescribe cross-sectional parameters independently of the locally computed stress field. The novelty of the present work lies specifically at this intersection. FEA-derived stress concentrations are fed back into the parametric design process to selectively modify local plate cross-sectional width within bounded, screw-hole-aware zones. A smoothstep taper is used to provide *C*^1^-continuous transitions. This approach differs from relying solely on fixed cross-sectional parameters, and from performing global material redistribution through topology optimization.

This article presents an update to a previously reported algorithm (21) and introduces a shape-adaptation feature for automated PSI design. The proposed algorithm analyzes the stress profile obtained from FEA. The proposed algorithm adaptively modifies the local plate cross-section at regions identified as having higher stress concentration. The resulting adaptive design is intended to reduce stress concentration and improve the mechanical reliability of the implant relative to a uniform-section baseline. FEA was performed to compare the mechanical behavior of the baseline and adaptive plate designs.

## 2 Materials and methods

The PSI design algorithm was originally discussed in our previous article, where we explained its basic framework. The baseline plate design includes marking the sweep path, identifying hole locations, and defining geometric parameters such as the plate’s width and thickness. After providing the required input to the algorithm, it automatically generates a 3D-printable CAD model in STL format.

In the present work, the developed geometry serves as the base configuration for a stress-informed, shape-adaptive PSI design algorithm. Based on the FEA output, the regions of higher von Mises stress concentrations were identified. Here, it is necessary to inform readers that a higher von Mises stress does not imply that the stress is beyond the yield limit. But it could be the user-defined threshold. In the present work, we mark the sections on the plate that experience elevated stress to validate the algorithm. The proposed algorithm locally changes the width and/or thickness of the plate in marked sections. A smooth taper was applied to preserve geometric continuity and manufacturability. The modified geometry was subsequently re-analyzed under identical boundary conditions, and the mechanical response of the adapted design was compared with that of the baseline plate.

The algorithm and FEA are explained as follows nomenclature used in this explanation is tabulated in **Table 1**.

**Table 1:** Symbols and definitions used in the proposed plate adaptation formulation.

Symbol	Definition
Γ	Three-dimensional centerline guide curve of the plate
*s*	Arc-length parameter along Γ, s∈[0,L]
*N*	Total number of sweep rectangles along the discretized curve
*R_k_*	*k*-th sweep rectangle
S	Ordered sequence of all sweep rectangles forming the plate
*h_k_*	Cross-sectional height (width-edge length) of rectangle *R_k_* [mm]
*t_k_*	Cross-sectional thickness of rectangle *R_k_* [mm]
${\hat{c}}_{k}$	Centroid of rectangle *R_k_*, ${\hat{c}}_{k}\in {\mathbb{R}}^{3}$
$u_{k}^{w},u_{k}^{t}$	Local unit vectors for the width and thickness edges of *R_k_*
*w_d_*	Nominal plate width—user design parameter [mm]
*T_k_*	Nominal plate thickness—user design parameter [mm]
$w_{0}=w_{d}/2$	Nominal half-width [mm]
$t_{0}=T_{k}/2$	Nominal half-thickness [mm]
λ_eq_	Target scale vector $\left[\lambda^{w},\lambda^{t}\right]$ applied to rectangles with $h_{k}\approx w_{d}$
λ_gt_	Target scale vector $\left[\lambda^{w},\lambda^{t}\right]$ applied to rectangles with $h_{k}>w_{d}$
*ϵ*	Geometric tolerance for the height equality check [mm]
*h*_max_	Maximum cross-sectional height within the core adaptation zone
*α_k_*	Spatial taper influence function, $\alpha_{k}\in \left[0,1\right]$
*ρ*	Transition range—number of rectangles over which taper is applied
*ϕ*(*τ*)	Cubic smoothstep kernel
dist(k,ℐ)	Discrete distance from rectangle *k* to the boundary of the core zone ℐ
$ℐ=\left[k_{a},k_{b}\right]$	Core adaptation zone index interval
**q***_a_*, **q***_b_*	3-D coordinates marking the region boundaries
*k_a_*, *k_b_*	Indices of the rectangles nearest to **q***_a_* and **q***_b_*, respectively
$\lambda_{k}^{\text{eff}}$	Effective (blended) scale vector applied to rectangle *k*
$\Delta w_{k}$	Scaled half-width of rectangle *k* after adaptation [mm]
$\Delta t_{k}$	Scaled half-thickness of rectangle *k* after adaptation [mm]
*M*	Number of independent adaptation regions
$\alpha_{k}^{m}$	Taper function of region *m* evaluated at rectangle *k*
**h** *_j_*	Centre coordinate of the *j*-th screw hole, $h_{j}\in {\mathbb{R}}^{3}$
*r*	Rounding radius [mm]

Bold symbols denote vectors (position, direction, or scale vectors).

### 2.1 Geometry of the PSI

The geometry of the PSI depends on the centerline guide curve that could be denoted by $\text{Γ}\subset {\mathbb{R}}^{3}$.This curve is parametrized by arc length *s* ∈ [0*,L*], where *L* is the total plate length. A rectangular cross-section $\text{Ω}\left(s\right)\subset {\mathbb{R}}^{2}$ is defined at each point **c**(*s*) ∈ Γ of the guide curve. The rectangular cross-section lies in the local Frenet–Serret frame {**T**(*s*),**N**(*s*),**B**(*s*)}, where **T** represents the unit tangent that lies in the direction of the orientation of the curve, **N** is the principal normal indicating the direction the curve is turning, and **B** = **T** × **N** is the binormal. Then the solid body P of PSI will be the swept volume as follows (refer Equation 1):

(1) P={c(s)+η N(s)+ζ B(s)|s∈[0,L], (η,ζ)∈Ω(s)}

The nominal cross-section Ω_0_ has half-width *w*_0_ and half-thickness *t*_0_ (such that the full nominal plate width and thickness are 2*w*_0_ and 2*t*_0_, respectively); these parameters are user-defined and govern the base geometry of the PSI. The four vertices that form the rectangular cross-section in the local frame are given by Equation 2:

(2) $$p_{\text{i}}\left(s\right)=c\left(s\right)+\eta_{i}\;N\left(s\right)+\zeta_{i}\;B\left(s\right),\;i\in \left\{1,2,3,4\right\}$$

where


$$\left(\eta_{1},\zeta_{1}\right)=\left(-t_{0},\;w_{0}\right),$$

$$\left(\eta_{2},\zeta_{2}\right)=\left(-t_{0},-w_{0}\right),$$

$$\left(\eta_{3},\zeta_{3}\right)=\left(t_{0},-w_{0}\right),$$

$$\left(\eta_{4},\zeta_{4}\right)=\left(t_{0},\;w_{0}\right).$$

The four vertices form a discrete geometric primitive, known as a sweep rectangle. This configuration contributes to the overall plate geometry, which can be represented as an ordered sequence.

(3) $$S={\left\{R_{k}\right\}}_{k=1}^{N},\;R_{k}={\left\{p_{i}\left(s_{k}\right)\right\}}_{i=1}^{4}\in {\mathbb{R}}^{4\times 3}$$

In Equation 3, *N* is the number of rectangles that form the cross-section of the PSI. These rectangles are placed along the discretized curve rectangular cross sections where *N* is the total number of cross-sectional rectangles along the discretized curve, and *s_k_*denotes the arc-length position of the *k*-th section.

### 2.2 Screw-hole placement

After creating the surface model of the PSI P by sweeping the rectangles S along the guide curve Γ, screw holes are placed. Screw hole positions must be clinically determined along the guide curve Γ. The center of the screw hole is marked by the surgeon from preoperative imaging data. Aside from the marked holes, two holes at each end of the plate are automatically positioned. This is to ensure secure fixation of the plate ends.

For automatic placement of the holes, the algorithm selects one point at each end, at an arc length of *r* (rounding radius). This ensures a margin around each end-hole. The positioning of the end holes can be represented by Equation 4:

(4) $$h_{\text{end}}=c\left(s^{*}\right),\;\;s^{*}=\min\{s:\ell \left(0,s\right)\geq r\}$$

End holes are created at both ends of the plate. This condition ensures that the end holes are not positioned too close to the plate’s end tip, which may affect the margin around the hole. The margin around the hole supports the plate’s structural rigidity, resisting pull-out forces under masticatory loading.

Clinically determined hole position is snapped to the nearest point on the sweep curve S, nearest neighbor search was used to perform this calculation.

(5) $$k_{j}^{*}=\arg\underset{k\in \left\{1,\ldots ,N\right\}}{\min}{\left\|{\bar{c}}_{k}-h_{j}\right\|}_{2},\;{\bar{c}}_{k}=\frac{1}{4}\sum_{i}p_{i}\left(s_{k}\right)$$

In Equation 5

${\bar{c}}_{k}$ is the centroid of the *k*-th sweep rectangle. This snapping operation aligns the hole axis with the local cross-section normal, ensuring that the drilled cylinder is always perpendicular to the plate surface at the hole location. This alignment is essential for proper countersunk screw seating.

The holes are created by subtracting a cylindrical geometry from the plate’s surface model; this forms a sharp annular edge around the hole’s perimeter. A sharp edge may increase local stress concentration under cyclic masticatory loads and create mechanical incompatibility with the sharp tip of a countersunk screw. To address this issue, a gradual, smooth, tapered transition has been created around the edge of each hole.

### 2.3 FEA of the PSI

FEA was performed on the baseline plate that was designed using the old algorithm Nayak et al. (21). The FEA simulation helps to understand the mechanical behavior of the PSI under loading conditions. The model incorporated material properties, boundary conditions, and muscle force loading representative of mandibular function refer **Figure 1**.

The CAD model of the PSI and the resected segment with the fibula flap are assembled in SolidWorks. The assembly is then saved in a neutral file format (.xt). This CAD model is subsequently imported into ANSYS Mechanical Workbench to simulate the right molar clenching. SOLID187 elements are used for meshing. An element size of 2.00mm is assigned to the bone, 1.0 mm to the PSI, and 0.5 mm to the screw. In this model, the screws are represented as a simple cylindrical shaft with a head, rather than a fully threaded screw. This assumption is to simplify the geometry. A mesh convergence study was performed to ensure the reliability of the reported stress results and to establish mesh independence prior to the main analysis. The mesh convergence was performed in ANSYS Workbench using the convergence tool. The mesh density was progressively refined, and the equivalent (von-Mises) stress at the critical region of interest was monitored at each stage. Refinement from 127,947 nodes (78,277 elements) to 144,521 nodes (89,103 elements) resulted in a change in peak equivalent stress from 303.83 MPa to 305.01 MPa, corresponding to a variation of 0.39%. As this change was well within the acceptable convergence tolerance, the finer-mesh configuration was adopted for all subsequent simulations reported in this study, ensuring that the results are not influenced by mesh density.

For model simplification, the mesh is assumed to be homogeneous, isotropic, and linearly elastic. PSI and screw were assigned the standard material properties of Ti-6Al-4V available in the ANSYS material library. At the same time, the bone part, including the reconstructed mandible, was modeled to be a dense cortical bone having Young’s modulus 13,700MPa and Poisson’s ratio 0.3 based on considerations as indicated by Shi et al. (22)Shi et al. (22). Bonded contacts were used to model the interface between the reconstruction plate, screws, and mandibular bone to simplify the representation of the mechanical fixation provided by the screws within the assembled finite-element model (9). Although the screws used in the present reconstruction were non-locking screws, the bonded screw interfaces were adopted as a modeling idealization to represent effective load transfer between the screws, plate, and bone rather than to reproduce the detailed thread-level mechanics of the screw–bone interface Wang et al. (23).

Muscle forces used to simulate right molar clenching are applied as specified in **Table 2**. The magnitudes of these forces are adopted from the literature; however, the signs of the individual vector components have been adjusted to conform to the coordinate system employed in the present study (24–27). Because the left superficial masseter and deep masseter were detached during the surgical procedure, the forces generated by these muscles were not included in the analysis. For the right molar clenching condition, the condyles are fully constrained in the x, y, and z directions, and the right molar is modeled with a frictionless support that prevents displacement of the molar in the positive z-direction.

**Table 2:** Muscle force vectors applied for simulation the right molar clenching.

Side	Muscle name	*x* (N)	*y* (N)	*z* (N)
Right	Superficial Masseter (SMR)	−28.38	−57.44	121.19
	Deep Masseter (DMR)	−32.08	21.03	44.53
	Medial Pterygoid (MPR)	71.36	−54.77	116.14
	Anterior Temporalis (ATR)	−17.19	−5.07	113.96
	Middle Temporalis (MTR)	−14.01	31.55	52.81
	Posterior Temporalis (PTR)	−9.28	38.14	21.14
	Inferior Lateral Pterygoid (ILPR)	12.63	−15.17	−3.49
	Medial Pterygoid (MPL)	−50.97	−39.12	82.96
	Anterior Temporalis (ATL)	13.65	−4.03	90.54
Left	Middle Temporalis (MTL)	14.22	32.03	53.61
	Posterior Temporalis (PTL)	6.13	25.21	13.98
	Inferior Lateral Pterygoid (ILPL)	−27.35	−32.87	−7.56

**Figure 1:**
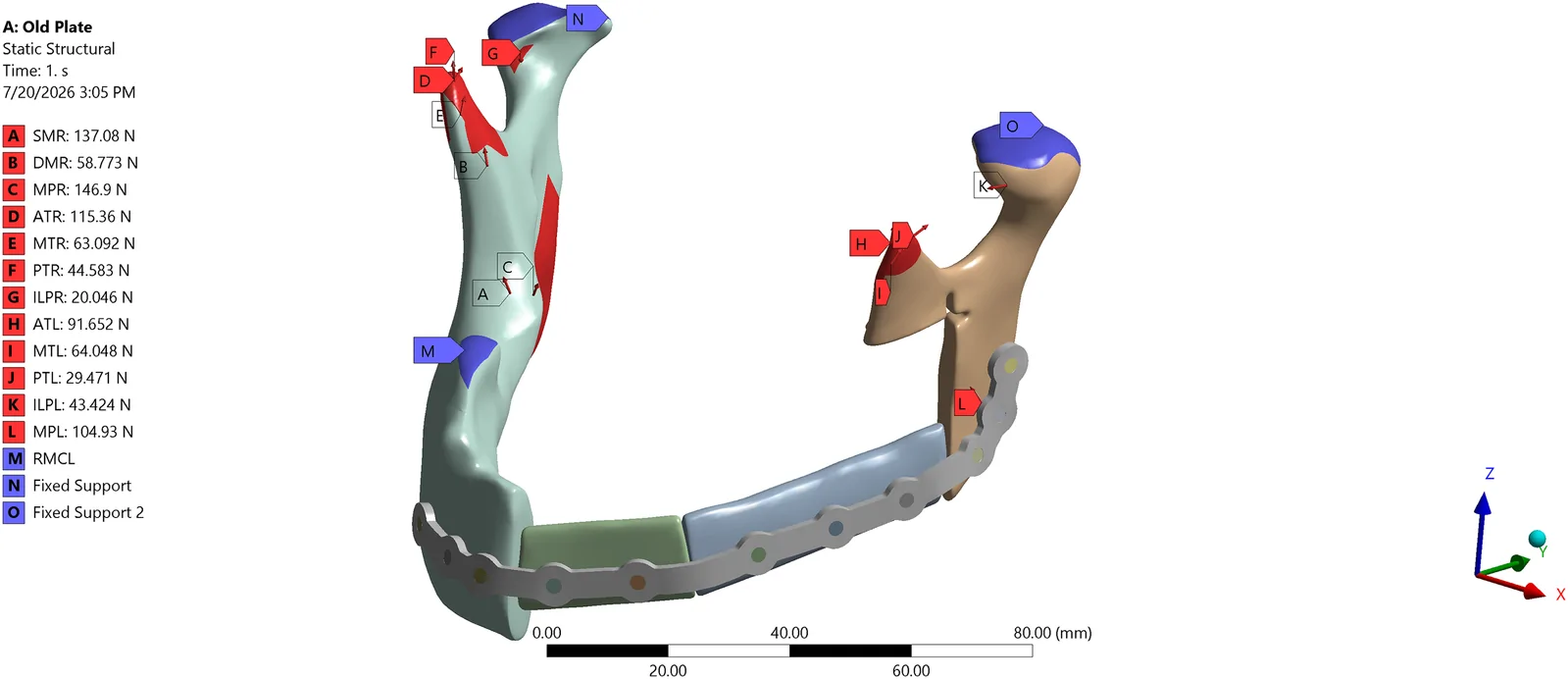
Muscle force direction and value included superficial masseter (SMR), deep masseter (DMR), medial pterygoid (MPR), anterior temporalis (ATR), middle temporalis (MTR), posterior temporalis (PTR), inferior lateral Ppterygoid (ILPR) for the right side of the mandible and medial pterygoid (MPL), anterior temporalis (ATL), middle temporalis (MTL), posterior temporalis (PTL), inferior lateral pterygoid (ILPL) for the left side of mandible, and RMCL showing the fixed support in normal direction of the molar teeth to simulate right molar clenching.

Based on the FEA, the region of stress concentration was identified. For this, the node coordinates and von Mises stress values from the baseline FEA were exported and processed in a custom MATLAB program. Each FE node was projected onto the nearest point on the plate’s centerline, giving every node a single arc-length position along the plate. The von Mises stress was binned along this arc length to obtain a stress-versus-arc-length profile (**Figure 2**). A zone was defined as any contiguous stretch of the plate along this profile in which the stress remained at or above a fixed threshold of 100 MPa. This threshold was selected to correspond to approximately one-third of the baseline global maximum stress (305.01 MPa). The zone was taken at the arc-length point where the profile crosses this threshold, with the threshold obtained by linear interpolation between adjacent bins. For better results, the boundary was pushed towards the nearest hole to avoid stress concentration near the cusp. This criterion identified two zones in the baseline analysis, corresponding to Zone 1 (holes 2–4) and Zone 2 (holes 8–10). The boundary coordinates $q_{a}^{m},\;q_{b}^{m}$ for each zone (Section 2.6) were taken directly from these threshold-crossing arc-length positions, mapped onto the plate centerline.

**Figure 2:**
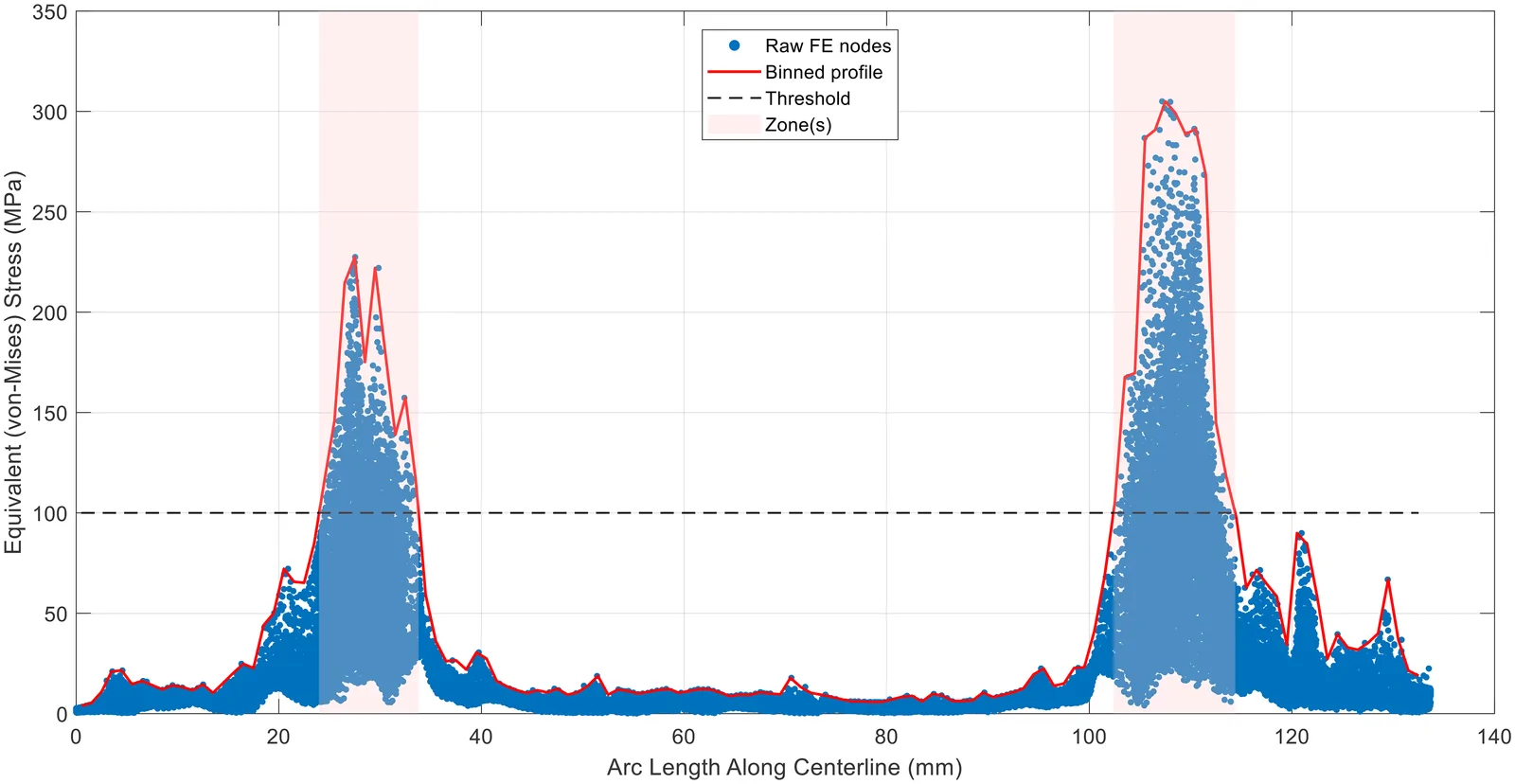
Von Mises stress profile along the arc length of the plate.

FEA is performed for each updated design. In the present research work, FEA has been conducted for the baseline plate and two independently generated design variants, referred to as Plate A and Plate B. The boundary conditions used in the FEA are kept identical to those described in Section 2.3.

### 2.4 Geometric scaling and width parameterization

After identifying the critical adaptation zone $ℐ=\left[k_{a},k_{b}\right]$ as discussed in the previous part, the algorithm modifies the geometry of the rectangles that fall under the adaptation zone and its surrounding taper region. The governing parameters for geometric modification of rectangles are computed from the geometry of the rectangle. These two governing parameters are the cross-sectional height *h_k_*and the spatial influence weight *α_k_*of the rectangle. The following section derives both quantities and explains how these quantities can combine to scale the geometry.

#### 2.4.1 Cross-sectional height measurement

The height of the *k*-th rectangle can be defined as the Euclidean length of the longer edge that links the first and second corner vertices refer (Equation 6).

(6) $$h_{k}={\left\|p_{2}\left(s_{k}\right)-p_{1}\left(s_{k}\right)\right\|}_{2}$$

The height of rectangles is computed for each rectangle that is placed along the sweep curve. This correctly captures the cross-sectional geometry of the plate with respect to the current iteration $S^{\left(n\right)}$. The measured heights ${\left\{h_{k}\right\}}_{k=1}^{N}$ provide an updated geometric characterization of the plate cross-sections before any scaling is applied.

The height *h_k_* represents the local width of the rectangular cross-section in the binormal direction **B**(*s_k_*), corresponding to the distance between the two vertices **p**_1_(*s_k_*) and **p**_2_(*s_k_*) that lie on the same edge of the rectangle. This measurement is essential for subsequent scaling operations, as it provides a baseline reference for determining the appropriate taper factors and maintaining geometric consistency across the entire plate length. Since the vertex coordinates are stored explicitly and updated after each geometric modification, the recomputation of *h_k_*ensures that all downstream calculations are performed on the most current plate geometry, thereby preserving accuracy throughout the iterative design process.

#### 2.4.2 Cross-section modification

The geometric response to the stress field is determined by classifying the measured height *h_k_*in relation to the nominal width *w_d_*. The logic behind this classification is that sections whose height matches *w_d_*(the intended design width) are considered to be in a geometrically nominal state and thus require one level of widening to alleviate stress. In contrast, sections that have exceeded *w_d_*potentially due to a previous adaptation cycle or a non-uniform sweep path already contain more material and are assigned a different, typically smaller scale factor. Meanwhile, sections below the threshold are considered structurally thin and left unmodified by default to prevent inadvertent bulges in non-critical areas.

Two scale vectors are user-defined:

-
$\lambda_{\text{eq}}=\left[\lambda_{\text{eq}}^{w},\lambda_{\text{eq}}^{t}\right]$ — applied to sections where $h_{k}\approx w_{d}$ (within tolerance *ϵ*).
-
$\lambda_{\text{gt}}=\left[\lambda_{\text{gt}}^{w},\lambda_{\text{gt}}^{t}\right]$ — applied to sections where $h_{k}>w_{d}$.

The per-rectangle target scale vector is therefore:

(7) $$\lambda_{k}^{\text{target}}=\{\begin{array}{ll} \lambda_{\text{eq}} & \text{if}\;\left|h_{k}-w_{d}\right|\leq \epsilon \\ \lambda_{\text{gt}} & \text{if}\;h_{k}>w_{d}\\ \left[1,\;1\right] & \text{otherwise} \end{array}$$

where *ϵ >* 0 is a user-defined geometric tolerance. Each component of the scale vector operates independently: the first component, λ*^w^*, scales the width edge (in the *h_k_*direction), while the second component, λ*^t^*, scales the thickness edge. However, the thickness is held constant throughout the study (see Constraint C2 in Section 2.7), implying that *λt* = 1. As a result, only λ*^w^* holds geometric significance.

#### 2.4.3 Uniform cross-section

The rectangle scale factor is calculated based on the other rectangles within the selected zone. Instead of varying the cross-section according to the stress profile, it maintains a uniform plate width. The system identifies the rectangle with the maximum height in the core zone and assigns its scale factor to each rectangle in that area, ensuring they all match the tallest rectangle’s height. This approach guarantees a consistent cross-section for the PSI.

(8) λkw=hmax  hk, hmax=max k∈ℐhk

Uniform cross-sectional width throughout the selected zone is achieved by selectively changing the height of sweep rectangles. This results in a more uniform load-bearing structure. This is particularly helpful when geometric variability causes stress concentration, as discussed in this article. For instance, variations in the curvature or torsion of the sweep path may cause certain cross-sections to become narrower than their neighboring sections, resulting in a localized reduction in plate width rather than a uniform deficiency across the entire plate. Sections outside the core zone are not included in the calculation of *h*_max_; their scaling continues to be determined by the spatial taper function described in the next section.

#### 2.4.4 Smooth spatial transition and *C*^1^-continuous width adaptation

The target scaling factor is applied to achieve cross-sectional modification of the PSI. The target scaling factor is a binary step function that ensures full scaling of the rectangles that fall under the core zone ℐ and no scaling outside of it. This would create an abrupt change in the cross-sectional width at the boundaries *k_a_*and *k_b_*. This discontinuity is undesirable because it introduces a sharp geometric transition on the plate surface, leading to stress concentration. A smooth transition of the cross-section could avoid this.

To prevent a sudden transition, we introduce a spatial influence function, denoted as *α_k_*: {1*,…,N*} → [0,1]. This function gradually shifts the target scaling from the core area to the neighboring unmodified 251 region over a user-defined transition length, *ρ*, which is measured in the number of rectangles.

(9) $$\alpha_{k}=\{\begin{array}{ll} 1, & k\in ℐ,\\ \phi \;\left(\frac{dist\;(k,ℐ)}{\rho }\right),\; & \text{dist }(k,ℐ)<\rho ,\\ 0, & \text{otherwise}. \end{array}$$

Here, the discrete distance from section *k* to the nearest boundary of the core zone is defined as

(10) $$\text{dist}\;\left(k,ℐ\right)=\min\left(\left|k-k_{a}\right|,\;\left|k-k_{b}\right|\right).$$

Equation 11 defines the cubic smoothstep kernel that governs the transition.

(11) $$\phi \left(\tau \right)={\left(1-\tau \right)}^{2}\left[3-2\left(1-\tau \right)\right],\;\;\tau =\frac{\text{dist}\left(k,ℐ\right)}{\rho },$$

where *τ* ∈ [0,1]. The resulting influence function smoothly decreases from unity at the boundary of the core zone to zero at the end of the transition region.

The smoothstep kernel is selected to satisfy the boundary conditions given in Equation 12:

(12) $$\phi \left(0\right)=1,\;\phi \left(1\right)=0,\;\phi^{\prime }\left(0\right)=\phi^{\prime }\left(1\right)=0.$$

Thus, the scaling of the rectangle up to a certain distance *ρ* from the boundary of the core zone is performed. The first derivative of the transition profile is zero at both ends. This ensures that the PSI does not have abrupt changes in cross-section at the core zone and that the adapted width profile is *C*^1^-continuous across the transition region.

In practical terms, *C*^1^ continuity ensures that the width of the plate connects smoothly at the transition boundaries, with a continuously varying rate of change. This prevents sharp geometric kinks that could lead to stress concentration.

The cubic smoothstep is preferable to linear interpolation, which only provides *C*^0^ continuity, and to a simple abrupt transition, which is entirely discontinuous. The cubic smoothstep enables a monotonic and symmetric transition while maintaining a slope of zero at both boundaries. This spatial taper allows the geometry to transition smoothly from the desired cross-sectional profile in the critical region to the original geometry outside that region, thereby enhancing both geometric regularity and the stability of the iterative adaptation process.

### 2.5 Coordinate transformation and rectangle reconstruction

Having established the target scale $\lambda_{k}^{\text{target}}$ and the spatial influence weight *α_k_*for every cross-section *R_k_*, we now derive the *effective* scale that is actually applied to the geometry. The key idea is straightforward: rather than switching abruptly between the full target and no change, we interpolate linearly between the two using *α_k_*as the blending weight. Formally, the effective scale vector is

(13) $$\lambda_{k}^{\text{eff}}=1+\left(\lambda_{k}^{\text{target}}-1\right)\cdot \alpha_{k}\in {\mathbb{R}}^{2},$$

where all arithmetic is performed component-wise. Equation 13 encodes three limiting cases that are worth making explicit:

- *α_k_*= 1 (rectangle lies inside the core adaptation zone ℐ): the effective scale equals the full target, $\lambda_{k}^{\text{eff}}=\lambda_{k}^{\text{target}}$.
- *α_k_*= 0 (rectangle lies outside the taper neighborhood): the effective scale is exactly [1, 1]^⊤^ and the rectangle is left entirely untouched.
- 0< *α_k<_* 1 (rectangle lies in the smoothstep transition zone): the effective scale interpolates smoothly between unity and the target, implementing the *C*^1^ taper.

This single equation therefore subsumes both the adaptation and the continuity enforcement into one compact expression.

#### 2.5.1 Local orthonormal frame of the cross-section

Applying a scale factor in three-dimensional space is non-trivial when the cross-sections are tilted, twisted, and translated along an arbitrary guide curve. A naive scaling of the global *x*-, *y*-, *z*-coordinates would produce sheared, non-rectangular cross-sections whenever the rectangle is not axis-aligned. The correct approach is to work entirely in the *local* coordinate frame of each rectangle.

We define two local unit vectors directly from the stored vertex coordinates of *R_k_* refer Equation 14:

(14) $$u_{k}^{w}=\frac{p_{2}\left(s_{k}\right)-p_{1}\left(s_{k}\right)}{{\left\|p_{2}\left(s_{k}\right)-p_{1}\left(s_{k}\right)\right\|}_{2}},\;u_{k}^{t}=\frac{p_{4}\left(s_{k}\right)-p_{1}\left(s_{k}\right)}{{\left\|p_{4}\left(s_{k}\right)-p_{1}\left(s_{k}\right)\right\|}_{2}},$$

where $u_{k}^{w}\in {\mathbb{R}}^{3}$ points along the *width* edge and $u_{k}^{t}\in {\mathbb{R}}^{3}$ points along the *thickness* edge. Because these vectors are derived from the *actual* vertex positions — rather than from the Frenet–Serret frame of the guide curve — they automatically encode every geometric transformation that has been applied to the cross-section: in-plane rotation to align with the curve tangent, out-of-plane tilt toward the second guide curve, and any prior scaling from a previous iteration. No additional book-keeping of rotation matrices is needed.

The centroid of rectangle *R_k_* is given by Equation 15:

(15) $${\hat{c}}_{k}=\frac{1}{4}\sum_{i=1}^{4}p_{i}\left(s_{k}\right)\in {\mathbb{R}}^{3}.$$

Together, the triple $\left\{{\hat{c}}_{k},\;u_{k}^{w},\;u_{k}^{t}\right\}$ constitutes a complete, frame-independent description of the rectangle’s position and orientation in space. Scaling in this frame is equivalent to stretching a unit square and then re-embedding it in ${\mathbb{R}}^{3}$ — a clean, invertible operation with no risk of shear or distortion.

#### 2.5.2 Scaled half-extents

Let $h_{k}={\left\|p_{2}\left(s_{k}\right)-p_{1}\left(s_{k}\right)\right\|}_{2}$ and $t_{k}={\left\|p_{4}\left(s_{k}\right)-p_{1}\left(s_{k}\right)\right\|}_{2}$ denote the current width-edge and thickness- edge lengths of rectangle *R_k_*, and let $\lambda_{k}^{w}$, $\lambda_{k}^{t}$ be the two components of $\lambda_{k}^{\text{eff}}$ from Equation 13. The *scaled half-extents* — the distances from the centroid to each edge midpoint in the adapted rectangle are calculated using the Equation 16:

(16) $$\text{Δ}w_{k}=\frac{h_{k}}{2}\lambda_{k}^{w},\;\;\text{Δ}t_{k}=\frac{t_{k}}{2}\lambda_{k}^{t}.$$

In the present study, Constraint (C2) fixes $\lambda_{k}^{t}=1$ for all *k*, so $\text{Δ}t_{k}=t_{k}/2$ is constant and the plate thickness is never perturbed. Consequently, the *only* degree of freedom that changes between different designs is the width half-extent Δ*w_k_*, which varies smoothly with both *α_k_*and the height classification. This single scalar per cross-section is what drives the entire stress-relief adaptation.

#### 2.5.3 Reconstruction of modified vertex positions

The four vertices of the modified rectangle ${\tilde{R}}_{k}$ are obtained by displacing from the centroid ${\hat{c}}_{k}$ along the local unit vectors by the scaled half-extents:

(17) $$\begin{array}{l} {\tilde{p}}_{1}\left(s_{k}\right)={\hat{c}}_{k}-\text{Δ}w_{k}\;u_{k}^{w}-\text{Δ}t_{k}\;u_{k}^{t},\\ {\tilde{p}}_{2}\left(s_{k}\right)={\hat{c}}_{k}+\text{Δ}w_{k}\;u_{k}^{w}-\text{Δ}t_{k}\;u_{k}^{t},\\ {\tilde{p}}_{3}\left(s_{k}\right)={\hat{c}}_{k}+\text{Δ}w_{k}\;u_{k}^{w}+\text{Δ}t_{k}\;u_{k}^{t},\\ {\tilde{p}}_{4}\left(s_{k}\right)={\hat{c}}_{k}-\text{Δ}w_{k}\;u_{k}^{w}+\text{Δ}t_{k}\;u_{k}^{t}. \end{array}$$

It is instructive to verify that Equation 17 preserves exactly three invariants that are required for correctness of both the mechanical model and the geometric pipeline:

1. Centroid invariance. Summing all four modified vertices and dividing by four immediately recovers ${\hat{c}}_{k}$, since the four signed displacement terms cancel in pairs. The plate centerline — and hence the load path along the mandible — is therefore not displaced by the adaptation.
2. Orientation invariance. The local frame $\left\{u_{k}^{w},\;u_{k}^{t}\right\}$ is recomputed from the *original* (pre-scaling) vertex positions and is not updated during the reconstruction. The transformation in Equation 17 is therefore a *pure stretch* in the local frame — no rotation is introduced, and the plate surface normal direction is preserved across all sweep sections.
3. Vertex-ordering invariance. Inspection of Equation 17 confirms that the counter-clockwise ordering ${\tilde{p}}_{1}\to {\tilde{p}}_{2}\to {\tilde{p}}_{3}\to {\tilde{p}}_{4}$ is preserved for any positive values of Δ*w_k_*and Δ*t_k_*. This ordering is a hard requirement of the downstream surface triangulation algorithm, which uses the signed cross product of consecutive edge vectors to determine face normal orientation; reversing the order would flip all surface normals and produce an inside-out mesh.

The three invariants collectively ensure that the adapted sweep sequence $\tilde{S}={\left\{{\tilde{R}}_{k}\right\}}_{k=1}^{N}$ is geometrically consistent, accurately represents the intended centerline, and can be readily used by the surface mesh generator and FEA pre-processor without the need for any additional repair or re-orientation steps.

### 2.6 Multi-region extension and overlap resolution

A single stress simulation often reveals more than one critical region, for example, stress concentrations near two different screw holes. The formulation is therefore extended to handle *M* ≥ 1 independent critical regions, each defined by its own coordinate pair $\left(q_{a}^{m},q_{b}^{m}\right)$. Each region m generates its own core zone, defined by Equation 18:

(18) $${ℐ}^{m}=\left[k_{a}^{m},k_{b}^{m}\right],$$

resolved by nearest-centroid search; taper profile $\alpha_{k}^{m}\in \left[0,1\right]$, computed via Equations 9, 10 independently;

target scale $\lambda_{k}^{m,\text{target}}$, classified per Equation 7 or Equation 8 independently;

• region final scale, defined by Equation 19:

(19) $$\lambda_{k}^{m,\text{eff}}=1+\left(\lambda_{k}^{m,\text{target}}-1\right)\alpha_{k}^{m},$$

• computed independently.

Where the taper zones of two or more regions overlap on the same rectangle *k*, a naive maximum scale rule would over-adapt the plate, while a minimum rule would under-adapt it. The algorithm instead resolves overlapping influences via alpha-weighted blending of the independently computed final scales:

(20) $$\lambda_{k}^{\text{comb}}=\frac{\sum_{m}\alpha_{k}^{m}\lambda_{k}^{m,\text{eff}}}{\sum_{m}\alpha_{k}^{m}},\;\text{if }\sum_{m}\alpha_{k}^{m}>0.$$

and

(21) $$\lambda_{k}^{\text{comb}}=1$$

for rectangles untouched by any region. The denominator $\sum_{m}\alpha_{k}^{m}$ is the sum of influence weights across all active regions at section *k*. A region whose core zone lies far from *k* (so $\alpha_{k}^{m}\to 0$) contributes negligibly to the blend; a region for which $k\in {ℐ}^{m}$ (so $\alpha_{k}^{m}=1$) contributes with full weight. The blended result therefore favors the more strongly active region naturally, without any *ad-hoc* priority assignment. When *M* = 1, Equation 20 reduces exactly to Equation 13, so the multi-region formulation is a strict generalization of the single-region case. Equation 21 ensures that the plate is modified only near the critical regions.

In the present study, the multi-region formulation was applied with *M* = 2 critical zones. The zones were identified from the FEA stress map of the baseline plate. Zone 1 was identified as the region between screw holes 2–4, and Zone 2 as the region between screw holes 8–10. Two design variants were subsequently generated to evaluate the effects of the proposed modifications at both zones. For simplicity, these variants are referred to as Plate A, in which the width adaptation defined by Equations 7-17 was applied only to Zone 1, and Plate B, in which the same adaptation was applied to both Zone 1 and Zone 2. Both variants retained the baseline plate thickness (1.6 mm), hole diameter (1.6 mm), and hole positions. In each adapted zone, the plate width was increased to 6 mm.

### 2.7 Manufacturability and geometric continuity constraints

The shape adaptation described in the previous sections is subject to certain constraints. These constraints are applied explicitly to each rectangle at every iteration, ensuring that the modified geometry remains mechanically feasible and suitable for processing by five-axis CNC milling or selective laser melting of titanium alloy.

(C1) Width bounds. The scaled half-width must remain within the admissible range throughout:

(22) $$w_{\min\;\;}\leq \text{Δ}w_{k}\leq w_{\max\;\;},\;\;\forall k\in \left\{1,\ldots ,N\right\}.$$

The lower bound *w*_min_ prevents the cross-section from becoming so narrow that the wall surrounding a screw hole loses structural integrity. The upper bound *w*_max_ prevents the plate from intruding into anatomical structures adjacent to the mandible Refer Equation 22.

(C2) Thickness preservation. Plate thickness is held invariant across all cross-sections and all adaptation iterations, as given by Equation 23:

(23) $$\lambda_{k}^{t}=1,\;\forall k\;\Rightarrow \;\text{Δ}t_{k}=t_{0}=\text{const}.$$

This constraint reflects the surgical requirement for a plate of known and uniform thickness that can be pre-bent intra-operatively.

(C3) Centroid invariance. All scaling is performed about the section centroid, as expressed in Equation 24:

(24) $${\tilde{c}}_{k}=c_{k},\;\forall k\in \left\{1,\ldots ,N\right\}.$$

This ensures that the plate centerline—and hence the load path along the mandible—is not displaced by the adaptation. It also prevents the adapted plate from drifting off the guide curve, which would invalidate the screw-hole positions computed in Section 2.6.

(C4) Taper regularity. The smoothstep kernel *ϕ* bounds the intersection width gradient in the transition zones:

(25) $$\left|\text{Δ}w_{k+1}-\text{Δ}w_{k}\right|\leq \delta_{\max},\;k\in \left[k_{a}-\rho ,k_{a}\right]\cup \left[k_{b},k_{b}+\rho \right].$$

The gradient bound *δ*_max_ is not an explicit input; it emerges from the choice of *ρ* and the target scale. Increasing *ρ* will reduce the maximum gradient and hence the severity of any geometric irregularity at the transition.

(C5) Orientation preservation. The local frame $\left\{u_{k}^{w},u_{k}^{t}\right\}$ is recomputed from the original (pre-scaling) vertex positions and is not updated during scaling, so the scaling operation is a pure stretch; it never rotates the frame:

(26) $${\tilde{u}}_{k}^{w}=u_{k}^{w},\;{\tilde{u}}_{k}^{t}=u_{k}^{t}.$$

This guarantees that the plate normal direction and surface tangent continuity are preserved across the sweep, which is a necessary condition for the downstream mesh generator to produce a valid, non-self-intersecting surface triangulation.

## 3 Results

The baseline PSI incorporates 11 screw holes, each 1.6 mm in diameter with a countersink to seat the screw head, and a uniform plate thickness of 1.6 mm. Its total volume is 1113.51 mm^3^, with a surface area of 1944.17 mm^2^. Based on the FEA results, two design variants were generated. Both variants retain the same thickness, hole diameter, and hole positioning as the baseline plate. The only modification occurs in two localized zones (zone 1 of holes 2–4 and zone 2 of holes 8-10), as shown in **Figure 3**, where the plate width was increased to 6 mm. Plate A has a volume of 1178.20 mm^3^ and a surface area of 2020.74 mm^2^, while Plate B has a volume of 1233.70 mm^3^ and a surface area of 2084.33 mm^2^.

**Figure 3:**
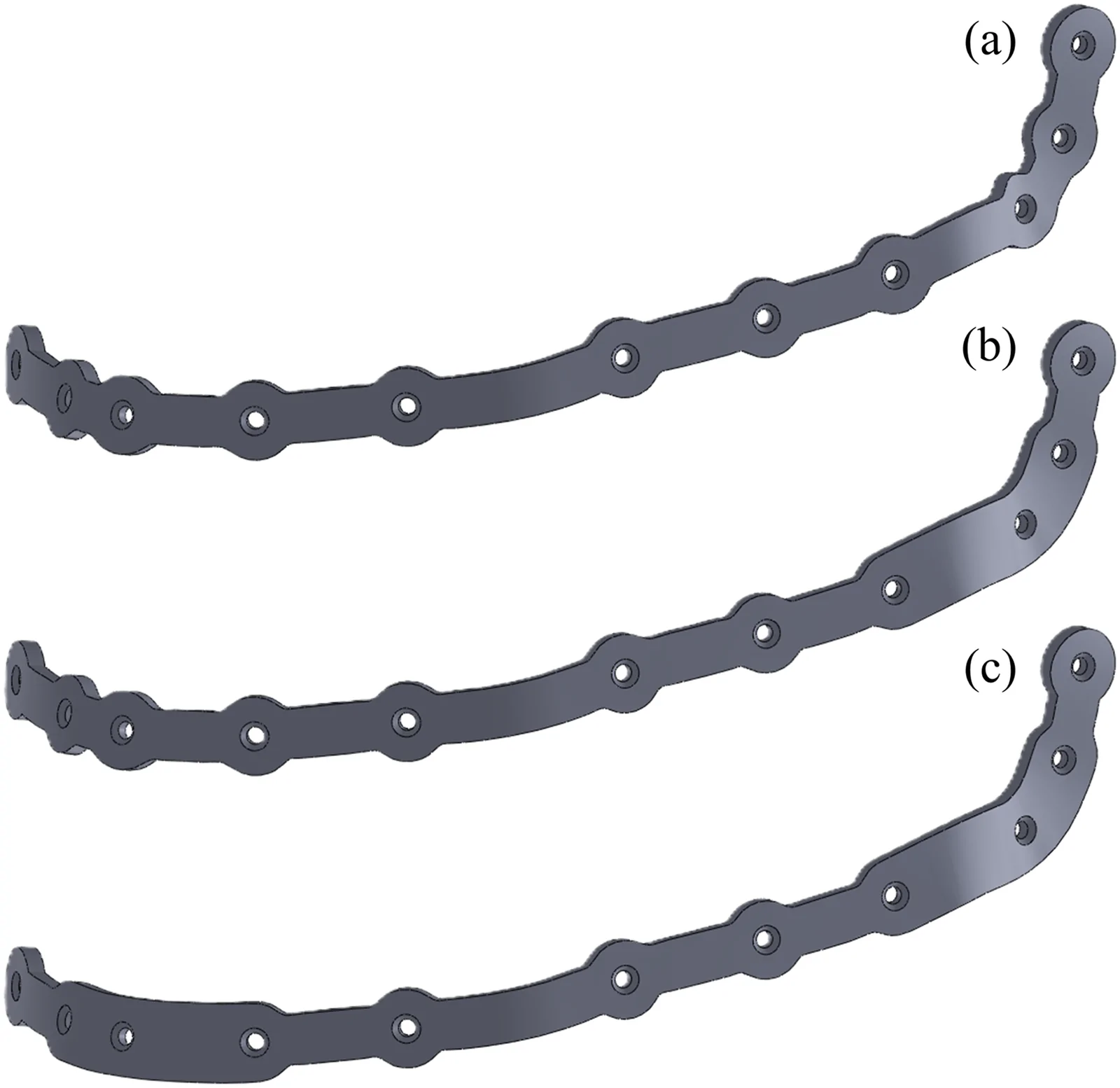
CAD model of **(a)** baseline plate, **(b)** plate with zone 1 modification, and **(c)** plate with zone 1 and zone 2 modifications.

**Table 3** summarizes the equivalent von Mises stress and equivalent elastic strain at Zones 1 and 2 for the baseline plate and the two modified designs. For the baseline configuration, the maximum von Mises stress in Zone 1 is 305.01 MPa with a corresponding strain of 0.0032, whereas in Zone 2 the von Mises stress is 220.77 MPa and the corresponding strain is 0.0021. The FEA of the baseline plate further indicates that the peak stress and strain are localized between holes 2 and 4 (Zone 1), as illustrated in **Figures 4a**, **5a**.

**Table 3:** Comparison of equivalent von Mises stress and equivalent elastic strain at zone 1 and zone 2 for the baseline plate, plate A, and plate B.

Parameter	Zone	Baseline Plate	Plate A	Plate B
von Mises stress (MPa)	Zone 1	305.01	195.20	204.64
	Zone 2	220.77	192.69	153.32
Equivalent elastic strain	Zone 1	0.0032	0.0018	0.0019
	Zone 2	0.0021	0.0018	0.0014

**Figure 4:**
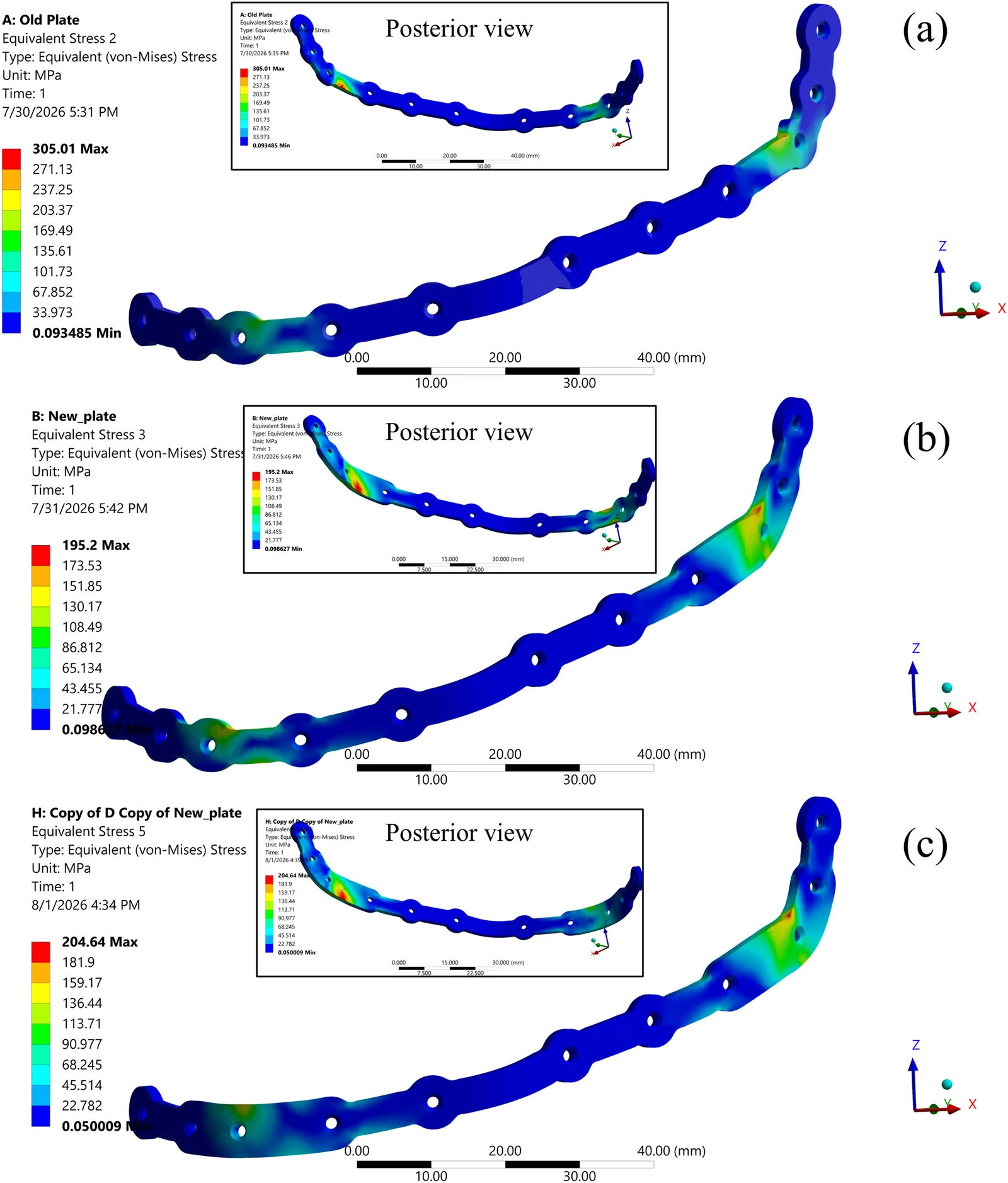
Von Mises stress distribution for **(a)** the baseline plate, **(b)** the modified plate after geometry modification at one critical zone, and **(c)** the modified plate after geometry modification at two critical zones.

**Figure 5:**
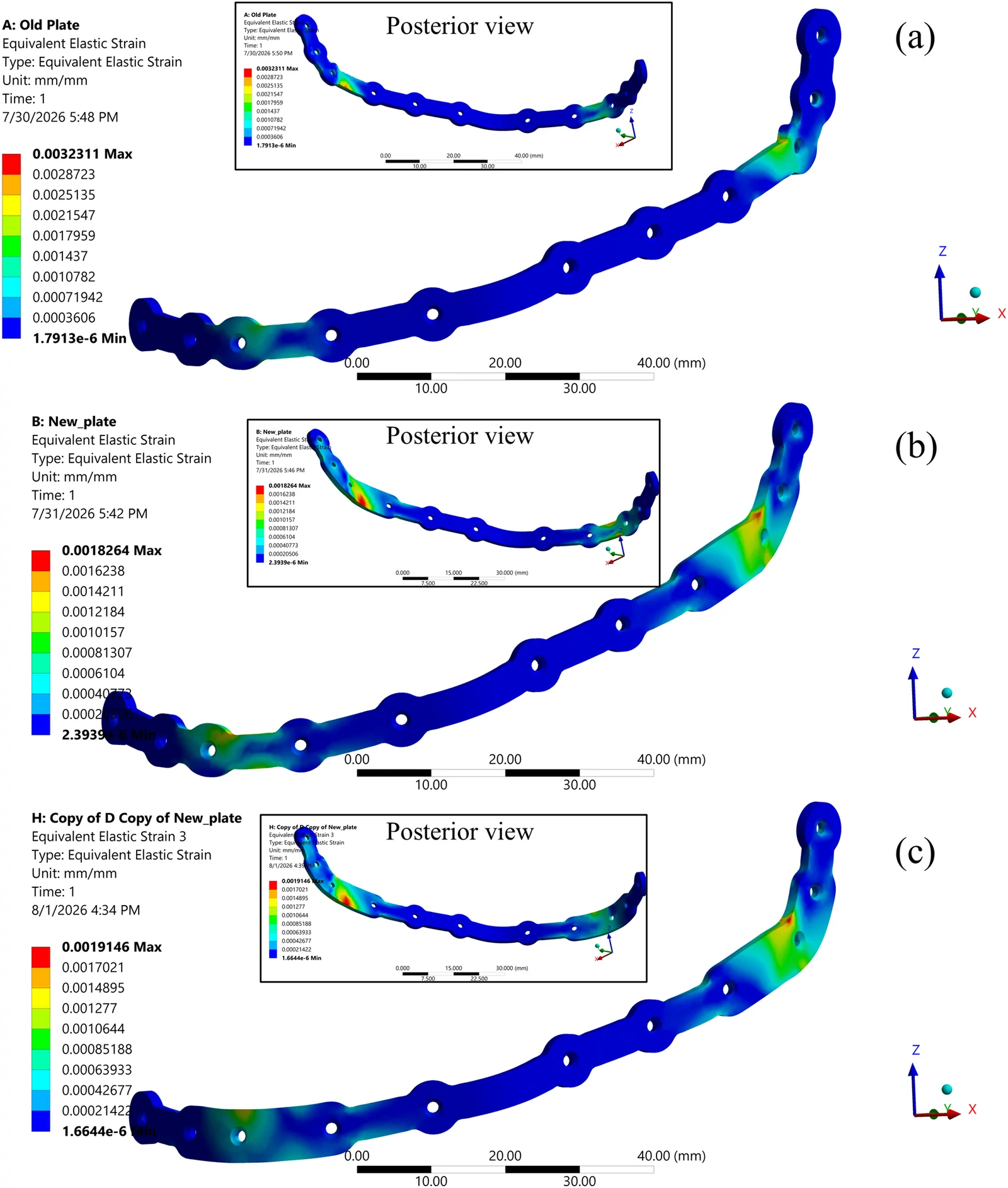
Equivalent elastic strain distribution for **(a)** the baseline plate, **(b)** the modified plate after one critical-zone geometry modification, and **(c)** the modified plate after two critical-zone geometry modifications.

**Figures 4a–c** and **5a–c** show the von Mises stress and equivalent elastic strain distributions across the entire plate for the baseline design, Plate A, and Plate B, respectively. These full-plate contour maps illustrate that no stress or strain concentration of comparable or greater magnitude than the Zone 1 and Zone 2 values reported in **Table 3** arose elsewhere on the plate as a result of the width adaptation.

In Plate A, modifications were made only to Zone 1, resulting in a 36.0% reduction in stress (down to 195.2 MPa) and a 43.8% reduction in strain (down to 0.0018) (**Figures 4b** and **5c**). Additionally, the stress and strain in the unmodified Zone 2 also decreased in Plate A by 12.7% and 14.3%, respectively. These changes indicate that local width adaptations help redistribute the load across the plate, affecting areas beyond just the modified region.

Plate B features adaptations in both Zone 1 and Zone 2. As a result, the stress and strain in Zone 2 were further reduced by 30.6% and 33.3%, respectively, compared to the baseline (**Figures 4c** and **5c**). However, a slight increase in Zone 1 stress and strain was observed relative to Plate A (stress: 195.20 to 204.64 MPa; strain: approximately 0.0018 to 0.0019). This represents a non-local mechanical trade-off between the two zones rather than a monotonic improvement from Plate A to Plate B: reinforcing Zone 2 further alters the plate’s global deflection and curvature profile, which increases the local bending moment, and hence stress, at Zone 1. Nevertheless, the stress in Zone 1 of Plate B remains 32.9% lower than the baseline value, indicating that simultaneous multi-region adaptation can achieve a favorable stress reduction at both critical locations without reintroducing baseline-level stress concentrations at either site. **Table 4** provides a summary of the percentage changes in stress and strain across Zone 1 and Zone 2 for all pairwise comparisons among the baseline plate, Plate A, and Plate B.

**Table 4:** Percentage change in equivalent von Mises stress and equivalent elastic strain at zone 1 and zone 2 across the baseline plate, plate A, and plate B.

Comparison	Zone 1 stress	Zone 2 stress	Zone 1 strain	Zone 2 strain
Plate A vs. Baseline	−36.0%	−12.7%	−43.8%	−14.3%
Plate B vs. Baseline	−32.9%	−30.6%	−40.6%	−33.3%
Plate B vs. Plate A	+4.8%	−20.4%	+5.6%	−22.2%

Negative values (−) indicate a reduction; positive values (+) indicate an increase, relative to the comparison stated in each row.

Both Plate A and Plate B were generated using the proposed algorithm, which uses FEA-derived stress information to guide modifications to the identified high-stress regions following analyst confirmation of the candidate zone. The successful adaptation across multiple regions demonstrates the proposed approach’s ability to accommodate multi-zone stress concentrations while reducing reliance on repeated manual design adjustments.

These results confirm that the algorithm can generate stress-adaptive PSI designs directly from FEA feedback, reducing the need for iterative manual adjustment.

## 4 Discussions

Designing of PSIs for mandibular reconstruction is a time-consuming process. In addition, the design iteration is challenging because the implant must exhibit complex freeform curvature to accurately match the patient’s facial contours. Such contours cannot be easily generated using conventional mechanical computer-aided design (CAD) tools and modeling approaches.

The proposed algorithm captures the facial contour and generates a corresponding CAD model in a few seconds. The algorithm’s parametric features ease the modification of design parameters. The parametric modeling facilitates efficient alignment of the final PSI design with the surgeon’s preoperative plan.

The algorithm produces an STL file that is ready for 3D printing. However, before printing and using the implant, its structural stability was evaluated. The maximum stress on the baseline plate was 305.01 MPa, well below the yield strength of the 3D-printed Ti–6Al–4V titanium alloy—524.2 MPa in the lowest case of electron beam melting (EBM). If the part were fabricated using selective laser melting (SLM), the yield strength could reach 1035.7 MPa (28). This indicates that the modelled elastic response of the baseline plate remained within the material’s yield limit under the simulated loading condition; as the present analysis considers a single static load case with idealized material and contact assumptions and does not assess fatigue, manufacturing variability, or screw loosening. However, to test the algorithm, regions with higher stress concentrations were targeted. It is a well-established fact that stress tends to concentrate at narrow cross-sections and curved geometric features. Similar observations are also noted in the baseline plate. In the baseline plate, stress was concentrated at the curvature connecting the left condyle to the fibula flap, as well as at the cusp of the margin around the 9th hole as shown in **Figure 4a**. Similarly, stress is concentrated in Zone 2, where the load is transmitted between the right resected part of the mandible and the reconstructed segment.

In the updated designs, reinforcement helps to reduce the stress concentration by increasing the cross- sectional area. The width of the plate can be increased or reduced in any proportion. But we kept it at 6mm to maintain the aesthetics. After strengthening zone 1, the stress is reduced as presented in **Tables 3**, **4**. On the other hand, in the case of Plate B, when zone 2 is also reinforced, the stress in Zone one is slightly increased compared to Plate A. The mechanical response of Plate B relative to Plate A reflects a non-local trade-off between the two adaptation zones, rather than a monotonic improvement across all reported metrics.

The comparison of the proposed algorithm with the topology optimization approach is an obvious question. In topology optimization, the weight of the plate is reduced by removing material from redundant areas. Such approaches typically involve repeated iterations between optimization and FEA, which may increase the computational requirements depending on the optimization settings and problem complexity (29). In contrast, the proposed approach uses a limited number of FEA-guided design modifications in the present implementation. However, a direct comparison of computational cost with topology optimization or conventional manual design was not performed in this study. Therefore, the relative computational efficiency of the proposed approach remains to be evaluated through future benchmarking.

Topology-optimized implants have been reported with better fatigue performance over conventional plates used in mandibular reconstruction (18). Topology-optimized implants typically require iterative sensitivity-based solvers and post-processing to correct numerical instabilities; in parallel, they also need to consider manufacturability constraints before the design can be validated and prepared (19). Then the plate is fabricated using Additive Manufacturing. It conservatively saves material and accounts for manufacturing constraints in the optimization. The highest amount of weight loss was 4.43% in one maxillofacial implant study with a margin of 4*µ*m (30). The adaptive scaling approach, on the other hand, recommends manufacturable, screw-hole-constrained geometry in a single FEA-driven feedback loop, offering a predictable design path at the expense of not exploiting the full unconstrained material-distribution space that topology optimization provides.

Some FEA-based studies of 3D-printed, topology-optimized implants have already incorporated mechanical validation and fatigue analysis (31, 32). On this basis, it is recognized that the present study reports a single static-load elastic analysis. Does not involve physical prototyping, mechanical testing, or fatigue. However, *in vivo*, the mandibular reconstruction plates experience cyclic masticatory loading. We thus think that such validation is significant for further research. This would help to bring the level of clinical evidence for this design approach closer to that found in these studies.

The present approach introduces reinforcement based on the stress profile while preserving the original screw holes and the implant’s overall curvature. The cubic smoothstep taper ensures a smooth geometric transition between the modified zones and the surrounding geometry, thereby avoiding abrupt stiffness changes that may arise from stepped width variations. Such abrupt changes in stiffness can act as secondary stress risers and potentially introduce new regions of stress concentration. Consequently, the proposed method enables zone-specific reinforcement to be incorporated into an existing patient-specific implant (PSI) design workflow without requiring complete re-derivation of the implant geometry.

The current algorithm is designed to handle only single-barrel reconstruction cases, so it cannot accommodate a branched PSI structure. The present study demonstrates reinforcement applied to two zones, evaluated as independent design variants (Plate A reinforcing one zone, Plate B reinforcing both). But this is not the algorithm’s limit; it can also reinforce multiple zones either simultaneously or sequentially.

The algorithm determines the reinforcement zones and adjusts parameters such as the updated width and thickness of the plate. However, we believe that simply automating this process will not guarantee better clinical outcomes. Therefore, key design parameters remain surgeon-specific, allowing surgeons to define them according to their unique clinical requirements. This approach strikes a balance between the mechanical performance of the implant and the surgical objectives.

There are also some other limitations of this study. Evaluation is based on a single patient-specific baseline reconstruction, under a single static loading condition (right molar clenching). The muscle forces were taken from published sources. The screws were simplified as cylindrical shafts with bonded contact. No mechanical or fatigue tests nor physical prototyping were conducted. The reported results must be taken with the above factors in mind.

## 4 Conclusion

In this study, an algorithm for FEA-guided, zone-based local width adaptation of a patient-specific mandibular reconstruction plate was proposed. It implements a cubic smooth-step zone-based local width adaptation function that is C^1^ continuous. FEA feedback from the baseline plate analysis was used to guide the adaptation. The width adaptation was applied to two zones where stress remained at or above a fixed threshold of 100 MPa. The algorithm generated two independent design variants (Plate A and Plate B) and reduced the peak von Mises stress and the equivalent elastic strain at both targeted zones relative to the baseline plate; the largest reduction observed was a 43.8% decrease in Zone 1 strain for Plate A. The algorithm required only a small number of FEA passes per design variant in this case study, which may translate to a reduced computational burden compared with topology optimization, though this has not been directly benchmarked. The design also preserved the predefined screw-hole locations and overall plate curvature throughout adaptation.

## Data Availability

The original contributions presented in the study are included in the article/supplementary material. Further inquiries can be directed to the corresponding authors.
